# Dr. Armstrong

**Published:** 1830-01

**Authors:** 


### OBITUARY.

We have to announce, with deep regret, the death of Dr. Armstrong, which took place at his house in Russell square, on Saturday, December 12th. Dr. Armstrong was well known to the profession for his indefatigable zeal. His peculiar opinions on the nature and treatment of fever, were perhaps, from his ardent enthusiasm, carried a little too far; but the doctrines he maintained upon this subject stamped him as a man of great ingenuity and original thought. As a lecturer on the practice of medicine, he was eminently successful; and, as a private practitioner, he enjoyed a large share of public confidence. By his professional brethren he was highly esteemed, and his loss will be deeply lamented.

---

